# Introductory Lecture to the Class of the Iowa University

**Published:** 1856-01

**Authors:** John R. Allen


### IOWA MEDICAL JOURNAL.

VOL. III. KEOKUK, IOWA, DEC. AND JAN., 1855-6. NO. I.

### ORIGINAL COMMUNICATIONS.

### [illegible]NTRODUCTORY LECTURE TO THE CLASS OF THE IOWA UNIVERSITY,

BY JOHN R. ALLEN, M. D.

GENTLEMEN: Prevailing custom demands at the hands of the Medical Faculty of this Institution, a public introductory, while proper courtesy to you, as students, requires some form of salutation.

As the organ of the Medical Faculty of the University of the State of Iowa, allow me thus formally to greet you, and to offer in their name, their congratulations and welcome to the halls of science; and at the outset of your course of instruction, to pledge you their most ardent efforts to advance you in the science of your adoption.

Next to Fourth of July orations, and Congressional speeches for Buncombe, perhaps few productions are more stale than medical introductory and valedictory addresses.

Eulogies upon the dignity and value of the profession are the themes of the one, while a solemn charge to the alumni, as to its deep responsibilities, and a few tears over the pangs of separation, render sadly dolorous the burthen of the other.

Now, I would in no way deny the propriety of these characteristics, feeling as I do, and think all brethren should, the full conviction of the dignity and deep responsibility of the profession, and I trust, all proper sensibility to the often strong personal ties which spring up between members of the same class, and between professor and student.

Indeed, outside of such matters, it is by no means easy to adapt an address of the kind to the circumstances of the case.

On such occasions, we are often complimented by the presence of a general audience, and if we should confine ourselves to the strictly professional, we become too technical for them; and if, on the other hand, we are purely popular, we sacrifice every feature which distinguishes the occasion.

A due regard, therefore, to you, gentlemen, and a proper respect for the non-professional here this evening to encourage you by their presence at your outset in a career in which they must necessarily be interested, demands of us something in which they and you have reciprocal interest. I propose then, for your consideration, *Man as the Subject of Medical Administration*.

W
1
10295J

A vast subject! truly, you will exclaim. Volumes have already been written, and many more are yet to follow upon it; and no doubt, when the human understanding in its most advanced state, shall have exhausted its powers in the investigation, much essential to its thorough understanding will remain forever locked up in the bosom of the Creator.

The subject involves no less than an inquiry into man's whole nature—all his relations as an organization, physical, vital, intellectual, and moral. How general, how faint the outline we can hope to draw!

In such an attempt, the first object of attraction is naturally the physical organization. Man is said by some, in the process of generation, up to full maturation, to pass successively through every grade of development, and at maturity to be the center of all animal organization—himself the head of animated nature.

Once a latent, amorphous, microscopic atom in the maternal stroma, he rises thro' every degree, evolving at every stage some new arrangement, until, in complete development, he stands the complicated microcosm of animal life, but superior to all whose type he may have assumed, in the ultimate superaddition of those higher and nobler qualities of mind, which distinguish him as superior to all the unnumbered tribes below him.

Into the minutiæ of this complex form, traced from its elementary components, up to a strange visceral organization and powerful mechanical arrangement, aroused into activity by the vital principle, are we called to look.

Thousands of scalpels have been plied by patient hands for centuries; Chemistry has heated her crucibles and applied her tests, and Microscopy lent her magnifying eyes, to trace and exhibit the hidden wonders of this diversified structure.

The more palpable objects of observation generally, have been for a long time, known. The bones and muscles have been numbered, named, and clearly described; the blood vessels followed to their remotest ramifications and anastomoses; the nerves, "those attenuated and gossamer threads of pearly tubuli," through whose agency the infinitely diversified frame is bound as by a chain of interstitial links, looping together every fiber, and by which every part becomes a minute whole, a variegated unit, have been followed, until lost in themselves; they form a filamentary and exquisitely sensitive envelope to the whole frame.

Not only so, but the more recondite elements have been detected; the molecular movements, globular measurements, and cellular developments, have been brought more or less to view.

But intricate and minute as are the investigations required for a proper acquaintance with the ultimate composition and compound materials of our bodies, one may hope from past discoveries, much future satisfaction.

But alas! a thorough knowledge of the most occult elements of organs and tissues; of their physical properties and chemical relations, gives as little insight into their physiological or vital functions, and much less of that intimate sympathetic association, which combines all into a unit so complete as .to leave no isolated part of its numerous members. We can learn little ot the office of any part, from a knowledge of its structure. Though we may discover the product or secretion of any viscus, and analyze its composition, we can neither explain the process or imitate its structure—every organ, every tissue has its own physiology, or certain vital actions and processes—and the life and health of each depend upon their proper exercise; and the sum of the harmonious sympathies and normal action of all is the life and health of the man.

We have, however, determined the offices of most of the organs of the body, in health, and are enabled to recognise the influence of disease, to some extent, upon them. But, so wide are the relations of the organization with things around it, when added to the diversified modifyidg causes inherent in a compound structure, the effort to prescribe remedies, on principle, becomes, to an honest mind, a matter of endless embarrassment.

A thousand extraneous causes modifying the cause and effects of diseases—a thousand internal peculiarities exert the same influences. Of the latter take the structure of parts, if you please, and what is seen? Some are obnoxious to certain forms of disorder, some to others—or the same form of morbid action, is thus greatly affected. An inflammation of one tissue of an organ, is very different in its course and effects from the same process in another tissue of the same organ. And how much more dissimilar when attacking organs widely separated in location, greatly differing in structure and function.

But while this is true, so intimate, complicated, and manifold are the physiological relations of our organization,—so closely associated are the most distant parts,—so readily and quickly do these parts act and re-act upon each other, that their reciprocal influences become incalculably multiplied, and before we proceed rationally to remove disorder of the one, we must estimate, as accurately as may be, the value of these mutual actions and re-actions.

It requires but a glance to convince us of the infinite variety of changes of function and structure to which so complex a system is subject, especially when disease is itself a monster, whose name is legion, and whose many phases are reciprocally multiplied, by the peculiarities of the organization upon which it acts.

Now there are certain unavoidable obstacles in the way of certain conclusions in determining the data for medical administration—such as ignorance of the essential nature of morbid agents, and still more, of what we term the vital principle. The latter is only known by its effects, it is intangible, inscrutable, and must invole in mystery, more or less complete, whatever it enters into as an element, and it is this which environs practical medicine at every step in difficulty.

In the physical sciences, we may often readily observe and classify facts and their relations; they are fixed and constant. But when life becomes an integral of any phenomenon, we can only, hypothetically calculate the results.

Our best ascertained facts are thus often disturbed by some deviation from what we supposed were settled conclusions, and hence a want of uniformity in the sequence of events, which constitutes the difficulty in determining medical facts.

Now if all diseases in all persons, exhibited the same series of results, and remedies used in the same diseases in all persons produced the same effects, we might be saved much of the embarrassment consequent upon their administration.

But the ever-varying influences of the peculiar conditions of the living principle, from inappreciable causes, as well as the more marked physical, mental and moral emotions, are so difficult of estimation that uncertainty must ever to some extent, mar the pretensions of medical science.

Now it will be remarked that the most natural and necessary changes of our systems work notable differences in the nature and treatment of disease.

The helpless infant clinging to its mother's breast is stricken by disease, unuttered but by signs of suffering, appealing, oh, how tenderly to our kindly offices peculiar to its tender structure.

Childhood in the hour of joy lies down with its peculiar inflictions, and turns with sickly distaste from the sports and toys of yesterday, and may soon drop as an unopened rose-bud among the withered leaves around it.

Youth, joyous in health, happy in the consciousness of present well-being, and hopes of future life and pleasure is blasted by the poisonous breath of the demon of disease, adapting his morbific agent to the expanding organization of this buoyant epoch.

Manhood, strong in the vigor of perfected development, exempt from many of the maladies of infancy, childhood, and age, is smitten in the strength of his might, by the still more vigorous arm of disease, and though he struggles as a strong gladiator, with his foe, succumbs at last to a force he can no longer resist.

Age, alas! itself a sure and deadly ill, needs no auxiliaries to do its work of death, but a regiment of accessories stand ready to aid and accelerate its inroads of decay, and hasten on the progress to darkness and the worm.

Such are some of the modifying conditions of the progress from birth to age, resulting from natural changes.

There are ten thousand other agencies no less fruitful in diversifying the human body in its relation to the use of remedies.

Think you, yon diminutive, frail and suffering infant, born in the confines of a city, of a fashionable mother—deformed by stays, enfeebled by indolence, softened by luxury, torpid from inactivity, and etiolated from shunning the genial sun light, identical in physical development and vital power, with the offspring of the vigorous country housewife, ruddy with health and ripe in development?

Think you, yon poor child with waxen skin, pearly eye, fragile form, and feeble motion, equal in bodily stamina, to the village boy, "with shining morning face,"—fat, rotund and active?

Think you, yon pale, slender and stooping young man toiling in the cavernous shades of the counting-house, or in the dust and smoke of a confined work-shop, to be compared in bodily strength and vital force with the sturdy yeoman, developed by salubrious toil, or the hardy mountaineer, ripened into manly stature and elastic action by the stirring chase and mountain breezes?

Think you that the man of wealth, and effeminate ease, resists with equal power, morbific agents, with the sturdy peasant who pays his rents?

Think you, that the man of business, absorbed in scenes of speculation, or harrassed by the perplexities of diversified transactions, equally exempt from disease or active in its combat, with the quiet agriculturist who waits in sober industry for seed time and harvest to reward his toils.

Think you, the professional man, confined to his books and briefs, incessant study, or, as with him of our own self-sacrificing avocation, exposed to breath of charnal house, to heat and cold, harrassed by annoying thoughts and active toil, who is seen to fade, so early, into the sear and golden leaf, comparable in brawny development, or bodily powers, with the mechanic who, by regular labor and alternate rest, sustains, to three-score years and ten, his healthy functions?

To these peculiarities, evolved by the natural epochs of our lives, or induced by habits in conflict with natural laws, we must add those strongly-marked, yet infinitely intermixed constitutional stamps, known as temperaments: distinguished by a predominance, more or less distinct, of some one of the systems or tissues of the organism.

These, when very well defined, stamp impressively their own characteristics, or if mingled, produce infinite diversity of organic susceptibilities or tinge with infinite variety the features of disease, and affect in unknown ways, the action of remedies.

But still we must superadd to this long list those hereditary maladies arising in those unfortunate persons, in whom nature, by some unknown departure from her generally beneficent care, or as a lasting infliction upon the licentious and bestial, combines elements, which generate, unhappily, those physical ills transmissable from parent to child.

Rheumatism thus prolongs its pains and fever—tortures its victims with aching agony, and sends them, often hobbling and decrepid to their doom.

Gout, the child of luxury and excess, thrusts daggers through the joints, and racking with intensest pain, stiffens the members, distorts the limbs, or, seizing some vital organ, wrings the life out.

Consumption thus vitiates the organic functions, blocks up with its chalky concretions, the vital passages, and sends its young, beautiful, and gifted victims, coughing, and parched with hectic fire, to a certain and early doom.

Scrofula, poisons with its purulent virus, the living streams and with bulging tumors, deep scars, and loathsome ulcers, disfigures, and melts down and wastes away the fair and goodly fabric.

Cancer, that promethian vulture, a hungry parasite, feeding on the frame by piece-meal. tortures, and by slow, but certain inroads, finds the lurking place of life, and undermines it.

Raving madness, that most terrific ill which flesh is heir to, replants its seeds of fire, to spring and burn afresh, in long generations.

Moody melancholy, its somber sister, stamps her heavy impress, to grow in sadness in succeeding minds.

Idiocy, the child of incest, is renewed in horrid lineaments, in unmeasured succession.

These are but a few of the physical agencies which, more or less, appreciably multiply the difficulties of comprehending man, as the subject of medical administration.

But we are not yet done with circumstances which most powerfully influence the organism in health and disease, and of vast value in practical medicine. We allude to mind, in its departments of intellect and passion.

Too apt are we to forget, in the complaints of men, of physical ills, the powerful agency of mind, in their production, and even in their cure.

We forget while considering mind as a function of matter, that it is only technically so—that while (except it be in the recent spiritual revealings,) it is nowhere observed, but in connection with matter, yet it is superior to it, or at least, exerts what no other product of an organ does, a voluntary, reactive agency over, not only its own organ, but upon the whole system. So powerful is this sometimes, as to enable man to say authoritively, he will or he will not be sick, and in accordance with this, resolves succumbs to, or successfully repell disease.

What is this agent? We know nothing of its essence. To mind, however, man owes his position among living creatures. This it is that originates, plans, and executes the schemes of life—this, that so distinguishes him by the display of powers, most wonderful—which renders him so prominently superior to all animals—this, that has led him from the wilds of barbarism, to the paths and refinements of civilized life,—this, which has elevated him to that throne of supremacy, before which all animated nature bows with reluctant, but coerced subserviency—this, in its moral department, which makes him a responsible agent, which claims for itself an independent life;—the something which "shrinks back on itself and shudders at destruction," —this, which informs him that he shall never die,—this, which claims a right to rule and dictate the body in its outward actions and to govern its inward emotions,—this, when properly influenced by moral and religious principles, which raises him to heaven, allies him to angels, and unites him to his God; or when debased and subdued by animal nature, sinks him below the beasts that perish, and assimilates him to demons.

Shall we then, in considering man as the subject of medical administration, fail to calculate the influence of this department of his constitution?—shall we forget his reasoning powers in disease, as medical philosophers, however,, he may fail to exercise them, as he too often does, in swallowing the nostrums of empyrics, or confiding in pretenders? Certainly not. Much less, again, can we disregard his moral faculties, or considering him as a being possessed of passions and emotions.

Our physical and moral natures are so mysteriously united, we can never hope to comprehend the mode of their union, and our bodily and moral infirmities are associated more intimately than we are likely to suspect—have a dependence which will often strike us with surprise, and reciprocal relations more active than we imagine.

Indeed! who can draw the line between real and imaginary woes—true and fancied sorrows—between afflictions from real extraneous causes operating immediately on the moral feelings, and those from some morbid state of the viscera, liver, stomach, nervous system---which equally, without producing obvious physical changes, so affect the mind, as to render it incapable of enjoyment, tho' surrounded by every social and personal means of indulgence, every object for which man longs and strives—amidst all, however, "that fixed and deadly gloom to which there is no sunshine in the summer sky, no verdure or blossom in the summer field, no kindness or affection, no purity in the very remembrance of innocence itself, no heaven, but hell, no God but a demon of wrath.

We are subject to passions and emotions highly pleasurable. We have others equally painful—we love, hope, and enjoy—we also hate, regret, and despair, and according to their nature and intensity, do they produce more or less marked physical phenomena.

Pleasurable passions cause a general expansion of vital action. "The blood flows freely through the extreme vessels, the countenance expands and brightens, and the whole surface acquires the glow and tint of health—the body feels buoyant and lively, and disposed to cheerful emotions, and every function is stimulated by the happy moral state."

"Love, hope, and joy," says Haller, "promote perspiration, quicken the pulse, augment the circulation, increase the appetite, and facilitate the cure of disease."

"Hope," says Sweetzer, "has been well termed a cordial, for what medicament have we so mild, so grateful, and at the same time so reviving? Its characteristic is to produce a salutary medium between every disorder and defect of operation, in every function, consequently it has the tendency to calm the troubled action of the vessels, to check and soothe the violent and irregular impetus of the nervous system, and to administer a beneficial stimulus to the oppressed and debilitated powers of nature.—How propitious such effects upon the various functions of the body.

But it must also be remembered that even these in excess, produce serious results—appoplexy, insanity, and even death has ensued. In their moderate exercise in preserving harmony between their indulgence, with bodily tranquilty, consist health and happiness, "leaving" as has been said, "extatic pleasures and rapturous feeling to creatures of a different nature."

The painful passions have a reverse influence, and enfeeble the vital functions. How many maladies owe their origin and persistence to morbid impression through them! How many chronic diseases consumptions, dyspepsias, and nervous disorders might be traced to moral causes, could we enter the sanctuary of hidden and inward feeling! How many a "worm in the bud," feed upon the elements of health, saps the vital energies, and hastens on, prematurely, decay and death! How many a young heart grows weak, and how many a form of beauty fades away, under the cold shadows of frowning fortune, none can tell.

Could we, in seeking the remote causes of disease, ferret out the secret troubles, the hidden pangs, which dwell about the heart, many ills now attributed to the ordinary causes of disease, bad diet, impure air, &c., would be referred to them.

In these days of stir and advancement, when mammon is the almost only god; when gain rules almost all the social relations; when man's nervous susceptibilities are exalted, and interests incessantly clash, what shocks and wounds must over-worked intellect and ardent passion encounter in the conflict of life? How staunch that frame which withstands, unscathed, the warfare,—how firm the mind which re-acts, unweakened from the concussions,—how stout the man, who each day renews the struggle, and feels no loss of general vigor!

Such are some of the more obscure effects of the depressing passions.

The more powerful ones often affect, more obviously the body, take hold upon the mind and strongly influence the frame.

Grief makes the heart to throb, the head to ache, the chest to heave, the voice to falter, appetite to fail, impairs every function, and exhaustion and inexpressible heaviness sinks to earth the languid frame—sobs its only utterance—tears its only relief.

> "What equal torment to the grief of mind
> And pining anguish hid in gentle heart
> That only feeds itself with thoughts unkind
> And nourisheth her own consuming smart!
> What medicine can any leech's art
> Yield such a sore, that doth the grievance hide
> And will to none her malady impart?"

Despair still more intensely depresses the powers and envelopes in unbroken gloom, the soul. "In it, the sources of comfort are closed up and no hope illumines the black clouds of the mind, no ray of joy pierces its thick darkness." The restless spirit seeks relief in reckless action, ceaseless dissipation, or self-destruction, or finds comfort in the bewildering commotion of raving madness, or the oblivious unconciousness of drivelling dementia.

> "He now no more, as once, delighted views
> Declining twilight melt in silverydews
> No more the moon a soothing lustre shows?
> To calm his fears and cheat him of his woes,
> But anguish drops from zephyr's fluttering wing.
> Veiled is the sun, and desolate the spring;
> The glittering rivers sadly seem to glide,
> And mental darkness showed creations pride."

So of anger, fear, and every other passion and emotion, we might show physiological effects, and trace an influence on disease. These are enough, however, for our purpose.

2

But, are you ready to ask, what of all this? What relation has it to the subject before us? Much, every way! The point we aim at, is to illustrate in a general way the complexity of our system, as a mechanical and vilta organization, and as a sentient being, subject to unnumbered impressions from surrounding agents, and as an intellectual and moral being affected in health and disease by the reciprocal influences of mind and body, and to show that we cannot as medical practitioners, leave out of view any of the conditions of health or any circumstances which affect the organism in any of its departments, in disease, which are appreciable without a dereliction of duty to the profession and to those who require our services.

And now, gentlemen, and my audience, what do you think of medicine? What think you should be the capacity and qualification of him who would dare administer medicine?

The best practitioner, even he who has had most advantages in the attainment of medical learning, as well as the largest practical experience, has to allow that he often finds himself greatly and not unfrequently completely confounded in determining his diagnosis and as to the administration of medicine.

How then is it possible that he who knows nothing of this wondrous organization, who never dreams of the unnumbered modifying influences which surround it, and are within, should presume to tamper with it.

"Man" says an old writer, "is a sort of musical instrument, and the strings of life and death are tuned on more keys than a Welch harp or a Scotch bagpipe, especially when an ill fiddler plays on his carcass; and that all men are ill fiddlers on their own carcass, and most abominable and jarring scrapers on those of others, unless they have devoted much time and judgement to acquiring a knowledge of the instrument."

Men therefore, ought to be very just in what they publish and assert, (or do,) in that tender and nice concern of life; for all the things in reference thereto, ought to be considered well, and treated with the greatest caution, for there lies no writ of error in the grave, but the sick man is finally concluded by the ignorance or knowledge of his physician.

How cautious, how discriminating, should be the Medical observer—what precision, what positiveness of diagnosis! How should he feel the maxim of Boussais, which he himself, however, forgot as a doctrinal writer, that "medicine is enriched only by facts." And how should he extort from the observer the exclamations used toward that great author by an accomplished critic: "What enlarged but cautious comprehensiveness in his general conclusions; what honesty and frankness in his admission of the frequent impotency of medical art! What an admirable tact and discrimination in his selection and use of remedial measures! How clear and sound the philosophy which illuminates and binds all this together! How true his appreciation of the emptiness and worthlessness of theoretical speculation!"

How few of the profession can claim as just, such a tribute to their powers, learning, or honesty! And even could they, from those competent to judge, it would be no guarantee to public appreciation.

The infamous pretender who never saw a scalpel, who knows neither the position nor function of an organ of the body, will concoct a compound, and by extensive advertising, and forged certificates, of victims snatched from the jaws of death, convince multitudes that he alone knows the hidden mysteries of the art; that his panacea will drive from earth every malady, and that men need no longer die from disease.

He contemptuously eschews all acquaintance with the intricaces of our organization, and discards, as unworthy his concern, all the efficient, modifying agencies, to which we have alluded. His nostrum meets all exigencies, and controls all contingencies. If regular doctors denounce him, it is from interested motives; if they refuse to use his remedy, it is for fear of its success; and so ignorance and empyricism are often triumphant, and sanctioned by a humbugged community; but it is but for a time. Disappointment brings back to reason, however. Science again becomes supreme—meekly waits her period again to be respected; again to be honored.

Among the ancients, the founders of medicine were deified, and temples erected to their honor, sacrifices offered on their altars, and oracles consulted at their portals, the highest expressions of a grateful people. The Æsclapiedæ were held in high estimation as benefactors of their kind.

The days of the worship of true medical heroes, however, are past, and he who, now-a-days, hopes for a temple may be thankful for a tomb. He who claims, "I am Sir Oracle," must allow the dogs to bark. He who has the temerity to enthrone himself as high priest of Æsculapias must expect his precepts to be disregarded, his athority derided, his wisdom unheeded.

Medical Appollos are now so numerous that worshippers fear lest they may be following strange gods.

Alopathia, though the lineal descendent of the gods has, not been allowed undisputed sway over the government of the medical art.

Various claimants dispute her rights. Antipathia, a medical Virago, whose maxims, like that of all bad-tempered women, is that contraires cure contraires, threatens her authority. Hydropathy, a strong-armed wench of the wash-tub, threatens to swarth her in cold sheets, and by the cold drouch or pelting shower-bath to chill her into submission, or by immersion strangle her to death. Madam Thompsonia, a ruddy old country woman, swearing and sweating, warns her by a whistle from her locomotive to clear the track, or a puff from her steam engine will blow her into "air—thin air." A very amiable pretender is in the person of a conciliatory and conservative young lady, Mdlle. Heterogenia Eclectica, who with less menacing aspect, smiles on her, but with words of kindness and claims of kinship, demands her portion of the heritage. And yet still an infinitesimal phenomenon, la petite Homœopathie, as brave as Jack the Giant Killer, threatens, with one decillionth of a grain of charcoal powder, leaving out the nitre and brimstone, to inflict such annoying pangs on her, as all her pills and potions cannot allay, nor all her anodynes alleviate.

Thus we are menaced, not only by individual upstarts and pretenders, but by medical systems, often as absurd in theory as they are inefficient in practice, sometimes, however, plausable and attractive.

But the science has thus far reacted from every assault. She has gone on observing nature, investigating her mysteries, and establishing facts. She is still unraveling the wonders of organization, and eliciting the most hidden vital processes. For centuries she has toiled, and to-day, with energy greater, and ardor warmer than at any former period. With a fervor unabated, her devotees are extorting facts from the most recondite functions. Induction has supplanted theory, truth its object, facts the evidence sought, and "there shall be hereafter amongst the lovers and seekers of truth, every where, a closer and more effective co-operation than has hitherto existed, in carrying forward, in their career of illimitable progress and indefinite improvement, all the branches of the great science of life."

What then does the contemplation of man in his medical relations, if I may so express it, involve?

It embraces, as we have said, an inquiry into his whole constitution, physical, vital, intellectual and moral.

It should view him in every relation into which this compound nature may bring him—it estimates the results of their actions and re-actions, in number, as yet unbounded, in variety and modes of action, in health and disease, uncalculated.

It includes the observation of disease in all its manifestations; a proper appreciation of the uniting sympathies of a complicated and heterogenious, yet harmonizing whole—it duly weighs the recuperative powers of the system; properly estimates the auxiliary powers of art, and rightly decides upon the time to interfere, the remedial agents to be used and the point at which we should forbear.

Who, then, is equal to the task? Certainly no one, fully. Our faculties are limited. All we can do is to bring every light of science to converge upon the subject, all the maxims of experience to bear upon each case; to invoke the combined indications of all appreciable surrounding circumstances, and upon bases thus derived erect the indications of cure, and then to select from the vast resources of the Materia Medica, other resources—such remedies as are presumed from a knowledge of their action, best to fulfil the ends proposed, then to leave results to a higher power than our own.

Thus, and thus alone can we fulfil the implied vows which we take in entering the profession; and thus, and thus alone, will we, as intelligent and honest practitioners, secure the only applause of true value, that of Heaven and our own.

In view of these considerations, what part have you to perform, my audience? Are you to forget the many difficulties and insurmountable obstacles to be encountered before a perfect knowledge of your wonderful organization, in all its relations can be obtained, in determining the true value of powers of the healing art. Or will you still turn like him who doses you with his catholicon, blindly, from the only source of light and yield yourselves the passive dupes to false systems and vain pretenders? Even admitting medicine to be a system of guessing, who, I ask you, is most prepared to guess correctly? The philosopher, the knave, or fool? If you prefer honesty, learning and experience, to charlantry and presumption in anything, surely it should be in the matter of health, of life and death, and I appeal but to your reason, when I demand your respect, ay, your regard and confidence, in regular medicine, honored by the voice of ages past, destined to an elevation far beyond the blind assaults of ignorance and presumption, the doubts of unstable incredulity, the sneers of vanity, or the ridicule of the epigramitist.

"Glorious their aim—to ease the laboring heart;
To war with death, and stop his flying dart;
To trace the source whence the fierce contest grew,
And life's short lease on easier terms renew;
To calm the frenzy of the burning brain;
To heal the tortures of imploring pain;
Or, when more powerful ills all efforts brave,
To ease the victim no device can save,
And smooth the stormy passage to the grave."

